# Ribonuclease D Processes a Small RNA Regulator of Multicellular Development in Myxobacteria

**DOI:** 10.3390/genes14051061

**Published:** 2023-05-09

**Authors:** Sarah M. Cossey, Gregory J. Velicer, Yuen-Tsu Nicco Yu

**Affiliations:** Institute for Integrative Biology, Department of Environmental Systems Science, ETH Zürich, 8092 Zurich, Switzerland; sarah.cossey@env.ethz.ch (S.M.C.); gregory.velicer@env.ethz.ch (G.J.V.)

**Keywords:** sRNA, ribonuclease, development, *Myxococcus xanthus*

## Abstract

By targeting mRNA transcripts, non-coding small RNAs (sRNAs) regulate the expression of genes governing a wide range of bacterial functions. In the social myxobacterium *Myxococcus xanthus*, the sRNA Pxr serves as a gatekeeper of the regulatory pathway controlling the life-cycle transition from vegetative growth to multicellular fruiting body development. When nutrients are abundant, Pxr prevents the initiation of the developmental program, but Pxr-mediated inhibition is alleviated when cells starve. To identify genes essential for Pxr function, a developmentally defective strain in which Pxr-mediated blockage of development is constitutively active (strain “OC”) was transposon-mutagenized to identify suppressor mutations that inactivate or bypass Pxr inhibition and thereby restore development. One of the four loci in which a transposon insertion restored development is *rnd*, encoding the Ribonuclease D protein (RNase D). RNase D is an exonuclease important for tRNA maturation. Here, we show that disruption of *rnd* abolishes the accumulation of Pxr-S, the product of Pxr processing from a longer precursor form (Pxr-L) and the active inhibitor of development. Additionally, the decrease in Pxr-S caused by *rnd* disruption was associated with increased accumulation primarily of a longer novel Pxr-specific transcript (Pxr-XL) rather than of Pxr-L. The introduction of a plasmid expressing *rnd* reverted cells back to OC-like phenotypes in development and Pxr accumulation, indicating that a lack of RNase D alone suppresses the developmental defect of OC. Moreover, an in vitro Pxr-processing assay demonstrated that RNase D processes Pxr-XL into Pxr-L; this implies that overall, Pxr sRNA maturation requires a sequential two-step processing. Collectively, our results indicate that a housekeeping ribonuclease plays a central role in a model form of microbial aggregative development. To our knowledge, this is the first evidence implicating RNase D in sRNA processing.

## 1. Introduction

All organisms modulate gene expression in response to changing environments. Similar to non-coding micro RNAs in eukaryotes, short sequences of non-coding RNAs known as small RNAs or sRNAs are important for such environmentally responsive gene regulation in bacteria [1]. The mechanisms by which sRNAs regulate gene expression are diverse and include the degradation of mRNA, protein sequestration, and modification of protein translation. Cis-encoded sRNAs can pair perfectly with their targets and typically alter gene expression by precluding ribosome binding but can also initiate mRNA degradation [2,3]. Alternatively, trans-encoded sRNAs have short sequences of complementarity that allow a single sRNA to affect many mRNAs by a variety of mechanisms [1,4]. Trans-encoded sRNAs typically require a cofactor, Hfq, to provide stability and promote interactions with their target mRNAs [5]. sRNAs can also indirectly affect gene expression by preventing regulatory proteins from interacting with their mRNA targets [6,7]. The majority of known sRNAs act as negative regulators [4] but some induce gene expression, for example by changing the secondary structure of mRNA upon binding to open ribosome binding sites and thereby allowing translation to proceed, or by stabilizing target RNAs [8,9].

sRNAs can act as primary regulators or work in concert with other regulatory machinery. In combination with transcription factors or other methods of gene regulation, sRNAs provide extra layers of control and help reduce leaky gene expression [10,11]. The speed at which sRNAs can change gene expression and their ability to prevent mRNA from being translated may allow cells to be more energetically efficient [10]. Rapidly and efficiently altering gene expression is especially useful during times of stress. In fact, many known regulatory RNAs alter gene expression in response to stressors such as starvation, oxidative stress, and envelope stress [12,13,14,15,16].

sRNAs are known to regulate genes that are involved in bacterial social behaviors. For example, PhrS stimulates quorum sensing in *Pseudomonas aeruginosa* [17]. CyaR sRNA negatively regulates *luxS*, resulting in decreased levels of quorum-sensing signaling molecules in *Escherichia coli* [18]. Similarly, Qrr sRNAs repress the transcriptional regulator of quorum sensing in *Vibrio cholerae* [19,20]. Biofilm formation in *E. coli* is also regulated, in part, by multiple sRNAs (McaS, OmrA, and OmrB) that control the operon responsible for encoding the structural components of curli filaments, a gene involved in regulating cellulose synthesis, and the adhesin poly-β-1,6-*N*-acetyl-d-glucosamine [15,21]. Interestingly, an sRNA also regulates the entry into the starvation-induced multicellular development program of *Myxococcus xanthus* [22].

**Figure 1:**
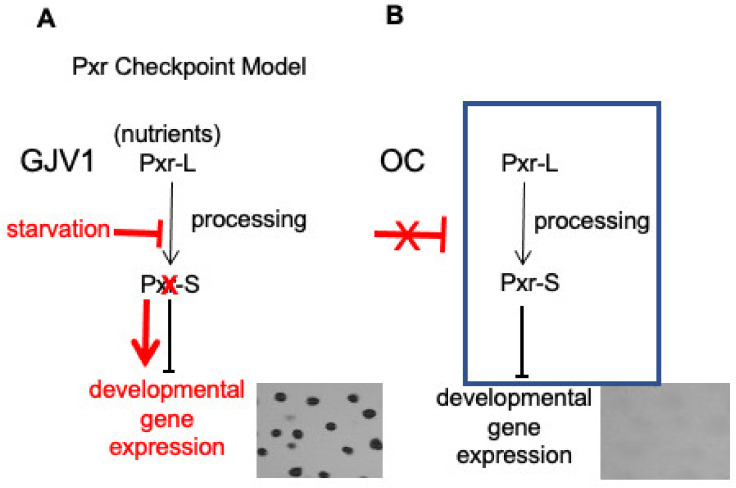
Checkpoint model of Pxr regulation of myxobacterial development. (**A**) When growing in the presence of nutrients, cells of the wild-type strain GJV1 produce Pxr-L sRNA, which is processed into a shorter form, Pxr-S. Pxr-S serves as the active checkpoint negative regulator and prevents entry into starvation-induced development. However, upon onset of starvation, blockage of sRNA processing rapidly eliminates accumulation of Pxr-S, thereby alleviating its inhibition of development. (The starvation pathway is shown in red.) (**B**) The developmentally defective strain OC is hypothesized to express Pxr-S constitutively due to lack of an early developmental signal needed to remove the checkpoint negative regulator.

*M. xanthus* is a Gram-negative soil bacterium that initiates a complex developmental program in response to starvation. Nutrient depletion activates the stringent response and initiates a cascade of signaling events culminating in the formation of spore-containing fruiting bodies [23]. A previous study identified an sRNA, Pxr, that negatively regulates *M. xanthus* development [22]. Pxr expression is dependent on a two-component histidine kinase/response regulator system, *pxrK* (*MXAN_1077*)/*pxrR* (*MXAN_1078*). *pxrR* encodes a σ^54^ response regulator upstream of the *pxr* promoter and is essential for Pxr synthesis, while the histidine kinase, *pxrK*, has a potential role in Pxr processing in addition to synthesis [24]. Pxr sRNA serves as a checkpoint to regulate the transition from vegetative growth to development [22]. Briefly, wild-type *M. xanthus* cells constitutively produce a large form of Pxr (Pxr-L), and when nutrients are plentiful, a smaller Pxr-specific form (Pxr-S) is also abundant. However, upon starvation, the accumulation of Pxr-S is rapidly diminished, and cells initiate their developmental program. We hypothesize that Pxr-S is processed from the longer Pxr-L transcript and acts as the negative regulator immediately blocking entry into development when nutrients are abundant (Figure 1A).

A developmentally defective strain named OC that evolved under constant vegetative growth conditions [25] was found to maintain high levels of Pxr-S for up to 24 h of starvation [22], suggesting that OC fails to transmit an early signal to remove the inhibitor (Figure 1B). Suppressor mutations that restore OC to developmental proficiency have so far been found exclusively in the *pxr* coding region or in genes demonstrated to control Pxr synthesis or processing [24]. The absence of functional Pxr appears to be essential for suppressing the OC phenotype. To further expand our search for genetic elements essential for the Pxr regulatory pathway, we conducted a screen of transposon-insertion mutants of OC and identified mutants that can undergo development [26]. We hypothesized that Tn insertions in genes important for the Pxr pathway will alleviate its negative regulation and allow cells to initiate fruiting-body formation. Indeed, several Tn mutants exhibited developmentally proficient phenotypes, including one designated TnE1. The TnE1 insertion locus has been mapped to *rnd* (*MXAN_5981*), which is predicted to encode Ribonuclease D (RNase D) [26]. A major function of the prokaryotic RNase D is to process the 3′ end of tRNA precursors [27,28].

In this study, we first test whether disruption of *rnd* per se restores developmental proficiency to the resulting mutant of OC. We then examine the role of RNase D in Pxr regulation with both in vivo complementation and in vitro processing assays. Finally, we develop a hypothetical model of the position and roles of RNase D in the overall regulatory pathway controlling the initiation of *M. xanthus* development.

## 2. Materials and Methods

### 2.1. Strains

The strain GJV1 was used as the laboratory wild-type in this study and was originally isolated from the wild-type strain DK1622 [29]. The two strains differ by only five mutations, and both are proficient in all social behaviors associated with *M. xanthus* [30]. GVB207.3—herein also referred to as “OC” for Obligate Cheater, ref. [22]—resulted from a long-term liquid selection experiment [25] during which it accumulated 14 mutations [30]. OC is unable to eliminate Pxr-S in response to starvation [22] and is therefore deficient in starvation-induced fruiting-body formation in pure culture. OC::TnE1 was found by screening a transposon-mutant library for restored developmental proficiency and has a transposon inserted in the gene *MXAN_5981* (*rnd*), a Ribonuclease D homolog [26]. GJV1::TnE1 and OC::TnE1-r were constructed by transforming a fresh growing strain of GJV1 and GVB207.3, respectively, with ~500 ng of OC::TnE1 genomic DNA and plated on CTT-kanamycin to select for clones resulting from a double crossover at the homologous regions flanking the transposon interstation site. All strains are listed in Table 1.

### 2.2. Plasmid and Strain Construction

Primers GV713 (TTTTCATATGCCGACCTACCCGCAAGGTGCCG) and GV714 (TCGCGGCCCCACCTTCACTTCAGGCG) were used to amplify a 1170 bp fragment of *rnd* (*MXAN*_*5981*) using Phusion DNA polymerase following manufacturer guidelines. To make pCR-*rnd*, the PCR fragment was ligated into the pCR-Blunt vector (Invitrogen). A vanillate-inducible *rnd* construct, pMR3629-*rnd,* was generated by ligating the excised EcoRI and NdeI-digested *rnd* fragment from pCR-*rnd* [31]. This plasmid integrates into a 1.38 kb region between positions 8498672 and 8500048 in the DK1622 genome that allows complementing *rnd* expression in the transposon mutant. The insertion in this region has no known negative impact on motility or fruiting body formation and sporulation [31].

Using pMR3629-*rnd*, strains bearing an inducible copy of *rnd* that complements the native, transposon-disrupted copy of *rnd* were constructed. Strain OC::TnE1 was grown to mid-log phase and transformed by electroporation with pMR3629-*rnd.* Clones resistant to both kanamycin and oxytetracycline were selected, yielding the strain OC::TnE1 pMR3629-*rnd.* Primers GV545 (CCCGGTCCGCTAGCCTGCCTCCC) and GV700 (GGTACGCGTAACGTTCGAATTC) were used to verify that pMR3629-*rnd* did not cross into the native *rnd* locus.

pBAD-his-*rnd* was made to overexpress the RNase D protein by amplifying an N-terminus histidine-tagged *rnd* gene with primers GV716 (TCTCGAGACATCATCATCATCATCATATGCCGACCTACCCGC) and M13r and using the plasmid pCR-*rnd* as a DNA template. The desired 1.3 kb PCR product was isolated from an agarose gel and digested with XhoI and EcoRI restriction enzymes. The gel-purified XhoI-EcoRI fragment was then ligated into an XhoI-EcoRI linearized vector pBAD-*Myc*-His to construct pBAD-his-*rnd*, which expresses a 6-histidine-tagged RNase D protein in *E. coli.* The constructed plasmid was also verified by sequencing.

To generate pCR-5982, which bears a truncated *MXAN*_5982 gene, an internal fragment of the *MXAN*_5982 gene was amplified using primers GV619 (TACACGATGACCCAGTTCCA) and GV620 (GACTCGTTGAGCCAGAGGTC) and cloned into the pCR-Blunt vector. The GJV1::*MXAN*_5982 mutant was constructed by transforming GJV1 with pCR-5982 and selecting for kanamycin-resistant colonies.

### 2.3. Developmental Assays

Proficiency at spore production during starvation was quantified. Strains were grown to mid-log phase in liquid CTT [32] at 32 °C and 300 rpm shaking, pelleted by centrifugation, and resuspended to a concentration of ~5 × 10^9^ cells/mL in liquid TPM [32]. Then, 50 µL of the cell suspension was spotted onto TPM (1.5 %) agar plates and TPM + 5 mM vanillate and incubated at 32 °C, 90% relative humidity (rH) for three days. Starved populations were harvested into 1 mL of ddH_2_0 using a sterile scalpel, heated at 50 °C for two hours, sonicated, and then dilution-plated into CTT (0.5%) agar. Colonies were counted after five days of incubation at 32 °C, 90% rH.

### 2.4. Northern Blot

Enriched small-sized RNA samples were isolated using the *mirVana* RNA isolation kit (Invitrogen by Thermo Fisher Scientific, Vilnius, Lithuania) according to manufacturer recommendations. Northern blot analysis was performed as described previously [22]. Briefly, samples were electrophoresed on a 10% UreaGel (National Diagnostics, Atlanta, GA, USA) followed by semi-dry transfer to a positively charged nylon membrane, BrightStar-Plus (Invitrogen by Thermo Fisher Scientific, Vilnius, Lithuania). Subsequently, the RNAs were UV-crosslinked to the membrane and hybridized with the biotin-tagged Pxr-specific probe overnight. Detection of Pxr was visualized with a Chemi-Doc MP image system (Bio-Rad, Hercules, CA, USA) after subjecting the blots to a chemiluminescent hybridization and detection assay (Thermo Scientific, Rockford, IL, USA).

### 2.5. RNase D Purification

The *E. coli* strain carrying the plasmid pBAD-his-*rnd* was inoculated from frozen stock into 5 mL LB medium and grown at 32 °C overnight. A 1:50 dilution of the overnight culture was generated in a 50 mL LB medium, and expression of RNase D driven by the pBAD promoter was induced by adding L-arabinose at a 0.1% concentration. A 1 mL bacterial culture was taken from the sample before the induction as well as from the sample after 3 h of induction at 32 °C to verify RNase D expression. To purify the N-6His-tagged RNase D, the cell lysate generated through sonication was subjected to an Ni-NTA agarose affinity column and the bound RNase D protein was eluted in 1 mL of elution buffer (Qiagen, Hilden, Germany). The expression and elution of the RNase D protein were confirmed by SDS-PAGE electrophoresis.

### 2.6. In Vitro Processing of Pxr by RNase D

Either 2 or 4 µL of the purified RNase D protein was mixed with 400 ng of enriched small-size RNAs isolated from strain OC::TnE1 (which only makes Pxr-XL) in a solution containing 0.5mM MgSO_4_ and 0.1mM ATP, and the resulting volume was adjusted to 7.5 µL using 10mM Tris-buffer (pH 8). The sample was then incubated on ice for one hour. The Pxr sRNA profile of the in vitro processed sample was analyzed by Northern blot as described above and compared to the detectable Pxr transcripts from RNA samples isolated from OC::TnE1 as well as the complementation mutant (OC::TnE1pMR3629-*rnd*) and the parental strain OC.

## 3. Results

### 3.1. The TnE1 Insertion in rnd Restores Developmental Proficiency

**Figure 2:**
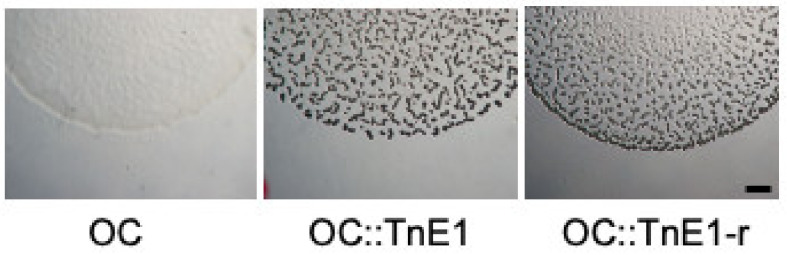
The transposon insertion TnE1 restores development in strain OC. The developmentally defective strain OC does not generate multicellular fruiting bodies upon starvation on TPM agar plates, while the originally isolated transposon-insertion mutant OC::TnE1 as well as the freshly re-constructed transposon-insertion strain OC::TnE1-r develop mature fruiting bodies. The images were taken three days after the onset of starvation. The scale bar is ~1 mm.

Our previous study found that the suppressor strain OC::TnE1 was able to form spore-bearing fruiting bodies despite its parental strain’s developmental defect [26]. Figure 2 shows the defect of the parental strain OC compared to the restored ability of OC::TnE1 to form fruiting bodies. To further verify that the insertion in *rnd* and not a second-site mutation suppresses developmental deficiency in the OC background, we transferred the TnE1 allele into a fresh OC background to reconstruct the transposon mutant (OC::TnE1-r). Indeed, the strain exhibits a developmental proficiency similar to that of the original transposon strain (Figure 2).

### 3.2. Disruption of rnd Is Sufficient to Rescue Development

**Figure 3:**
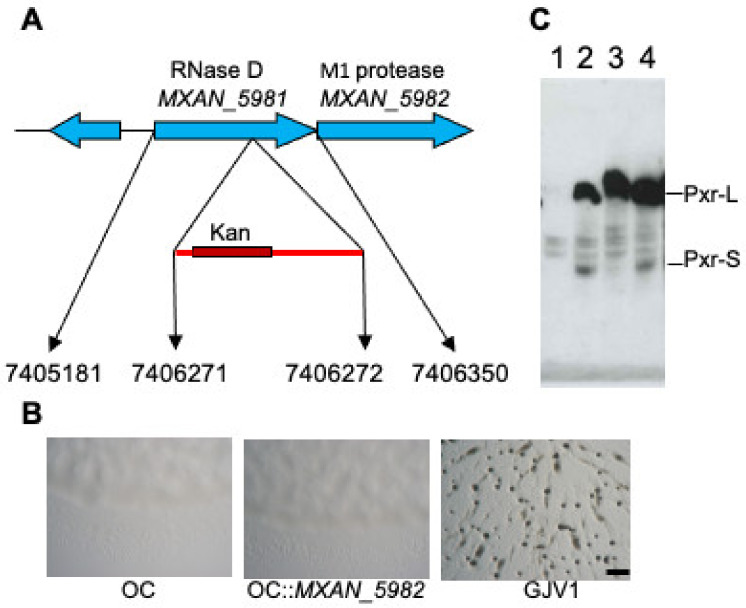
A transposon insertion in *rnd* affects Pxr sRNA processing. (**A**) The insertion of transposon TnE1 is mapped to the *MXAN_5981* (*rnd*) gene encoding an RNase D enzyme homolog. The diagram depicts the location of the putative transposon insertion site in the *rnd* locus. Numbers at the bottom of the figure indicate, from left to right, the genomic positions of the start of the *rnd* coding sequence and the sites adjacent to the inserted transposon and the end of the *rnd* gene (adapted from [26]). (**B**) Disruption of *MXAN_5982* in OC does not restore development. On starvation agar plates, OC fails to form multicellular fruiting bodies, while GJV1 forms darkened fruiting bodies within three days of starvation. (**C**) The *rnd* transposon mutation is associated with the absence of Pxr-L and Pxr-S. Northern blot assay showing the accumulation of the Pxr-specific transcripts from strains GJV1∆*pxr* (lane 1), DK5057 (*asgA* mutant, lane 2), GJV1::TnE1 (lane 3), and OC::TnF4 (a transposon mutant that makes normal Pxr-L and Pxr-S, lane 4) [26].

An analysis of OC::TnE1 revealed that the transposon insertion is located within *rnd* (*MXAN*_5981), suggesting that the insertion disrupts the function of Ribonuclease D. The *rnd* gene is part of an operon and is co-transcribed with the downstream gene *MXAN_5982*, which encodes an M1 peptidase homolog (Figure 3A adapted from [26]). A transposon insertion in an upstream locus might cause a detrimental polar effect on the expression of downstream genes. To verify that the restored developmental proficiency of the transposon mutant is not due to a defective *MXAN_5982*, we constructed OC::*MXAN_5982* by a plasmid interruption and assayed for fruiting body formation. As shown in Figure 3B, this mutant is unable to form fruiting bodies and appears morphologically similar to its developmentally defective parental strain OC. This result indicates that lack of a functional *rnd* and not a defect in *MXAN_5982* allows OC to undergo development. Previously, we proposed that *rnd* may be involved in processing Pxr into the active small form [26]. To test this hypothesis and to avoid potential effects of the 14 mutations in the OC background relative to GJV1 [30], we constructed GJV1::TnE1 by transferring the TnE1 allele into a GJV1 background (see Materials and Methods). Northern blot analysis of GJV1::TnE1 revealed that the *rnd* mutant did not have detectable levels of Pxr-S (Figure 3C), which is consistent with the hypothesis that RNase D is involved in Pxr processing. Furthermore, this result indicates that the lack of Pxr-S is likely the cause for the suppressor’s phenotype, allowing cells to bypass the Pxr-mediated developmental checkpoint. Interestingly, a novel Pxr transcript longer than the normal Pxr-L (Figure 3C) accumulates in the GJV1::TnE1 mutant; we name this longer transcript Pxr-XL.

### 3.3. Complementation of Defective rnd Prevents Development

**Figure 4:**
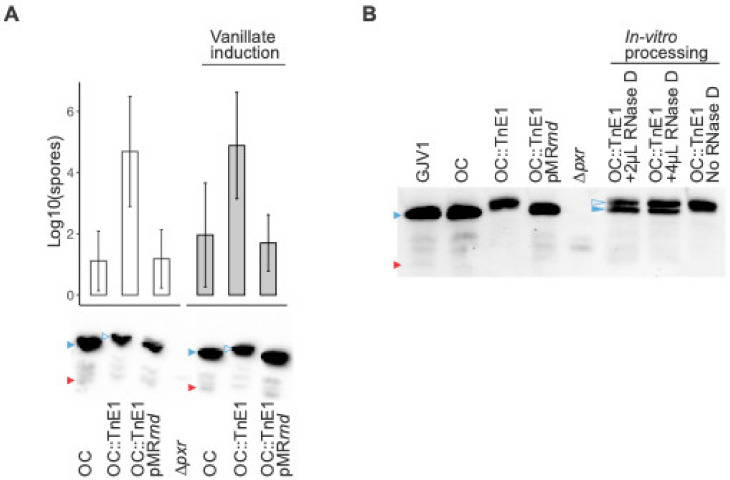
Wild-type *rnd* introduced into OC::TnE1 restores the OC developmental defect and processes Pxr sRNA in vivo and in vitro. (**A**) Top: Spore production of strains OC, OC::TnE1 and OC::TnE1pMR*rnd*. White and grey bars depict spore production of strains in the absence and presence of 0.5 mM vanillate, respectively. Error bars show 95% confidence intervals, *n* = 3 independent replicates. Bottom: Northern blot analysis of strains OC, OC::TnE1, and OC::TnE1pMR*rnd* in the absence (left) and presence (right) of 0.5 mM vanillate, as well as strain ∆*pxr* (middle, for which spore production is not shown). Blue triangles indicate Pxr-L, red triangles Pxr-S, and open blue triangles Pxr-XL. (**B**) Northern blot analysis of the in vitro processing assay of Pxr using purified RNase D. The Pxr profiles from the RNA samples isolated from respective strains are shown on the left side of the image, whereas the Pxr profiles of the RNA samples that went through the in vitro processing protocol either with or without purified RNase D are shown on the right side of the image. Blue triangles indicate Pxr-L, red triangles Pxr-S, and open blue triangles Pxr-XL.

To further verify that the restored developmental phenotype in OC::TnE1 is due to a defective *rnd*, we constructed OC::TnE1pMR3629-*rnd,* which expresses *rnd* under a vanillate-inducible promotor at a non-native locus [31]. If the developmental proficiency in OC::TnE1 is caused by the lack of functional RNase D, exogenous complementation of a wild-type *rnd* allele should result in a developmentally deficient phenotype similar to that of strain OC. Indeed, starved OC::TnE1pMR3629-*rnd* populations cannot develop properly and exhibit a sporulation defect similar to that of OC (Figure 4A). This result further demonstrates that the *rnd* mutation is solely responsible for the OC::TnE1 phenotype. As shown in Figure 4A, developmental deficiency was evident in OC::TnE1pMR3629-*rnd* even without vanillate induction, suggesting that the vanillate-inducible promotor is “leaky” and that a basal level of *rnd* expression in the “off” state is sufficient to block development.

### 3.4. Complementation of rnd Restores Pxr-L and Pxr-S Accumulation

Consistent with the Northern blot analysis of GJV1::TnE1 (Figure 3B), cells of OC::TnE1 accumulated the previously undescribed, larger species of Pxr sRNA (Pxr-XL) (Figure 4A). Complementation with a wild-type *rnd* allele resulted in the accumulation of Pxr-L and no detectable levels of Pxr-XL (Figure 4A), suggesting that RNase D is involved in the processing of Pxr-XL to Pxr-L. Because Pxr-XL has never been observed during either vegetative growth or development in any *M. xanthus* strains except in the *rnd* mutants reported here, we hypothesize that the existence of Pxr-XL is transient, and that Pxr-XL is processed into Pxr-L by RNase D very rapidly after it is made. The production of transcripts that are instantaneously processed into more stable or active forms is a common feature of eukaryotic miRNAs [33] but has not been frequently reported for bacterial sRNAs. The precise role of Pxr-XL in the Pxr pathway is not clear.

### 3.5. RNase D Processes Pxr-XL to Pxr-L In Vitro

In order to determine the precise role of *rnd* in Pxr processing, we carried out an in vitro assay by adding purified RNase D protein to enriched small-sized RNA samples from cells of OC::TnE1. If RNase D is responsible for both processing steps (XL to L and L to S), we would expect to see both Pxr-L and Pxr-S in the Northern blot assays. Figure 4B shows the clear processing of Pxr-XL to Pxr-L with the addition of RNase D, while only Pxr-XL is present in the sample without RNase D. The presence of Pxr-S, however, is not clear. Alternatively, RNase D may, in fact, process Pxr-L to Pxr-S, but Pxr-S may be unstable on its own and potentially require a partner for stabilization that is not present in our in vitro system.

## 4. Discussion

**Figure 5:**
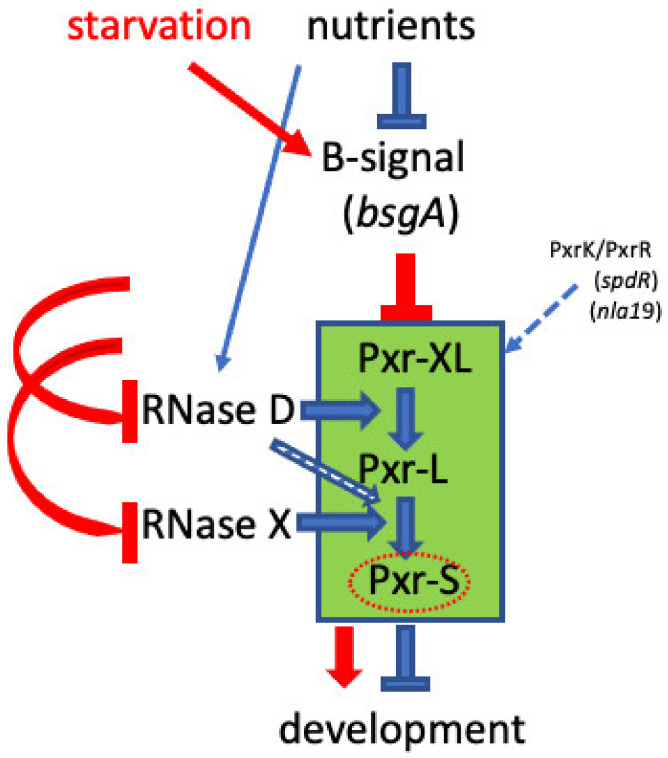
Hypothetical role of B-signaling in the Pxr sRNA checkpoint model. Epistatic effects of *rnd*, *pxrK,* and p*xrR* null mutations on a *bsgA* defect suggest that the Pxr-processing checkpoint is negatively controlled by the *bsgA*-mediated B signal. We hypothesize that when nutrients are abundant, B-signal is in an “off” state that allows Pxr processing to generate the developmental negative regulator Pxr-S. Upon starvation, however, we hypothesize that the B-signal is activated in a manner that blocks accumulation of Pxr-S by either reducing the production or stability of RNase D or inactivating a putative additional ribonuclease (designated RNase X in the diagram). Pathways during growth at high nutrient level and upon starvation are depicted in blue and red, respectively. Pxr-processing events are highlighted by the green rectangle. The open blue arrow indicates a possible role of RNase D in processing Pxr-L to Pxr-S, in contrast to the alternative hypothesis that a ribonuclease other than RNase D performs this function. The dashed blue line indicates the positive control of the PxrK/PxrR two-component system on Pxr sRNA synthesis.

Our previous study found that a transposon-insertion mutation localized in the *rnd* locus suppressed its parental strain’s (OC) developmental defect and allowed the mutant OC::TnE1 to undergo development and produce heat-resistant spores [26]. The *rnd* gene encodes a Ribonuclease D protein (RNase D), which is typically involved in bacterial tRNA maturation/processing [27,28]. Here, we demonstrated that a defective *rnd* is solely responsible for the suppressor’s phenotype and that RNase D can undertake a novel function as a bacterial sRNA processing enzyme. The introduction of a wild-type *rnd* allele into the mutant genetic background complemented the defective *rnd* and reversed the developmental phenotype back to that of the parental OC strain. The *rnd* mutation impairing RNase D production and/or function was associated with the absence of the developmental negative regulator Pxr-S sRNA (Figure 3B), offering a molecular basis for how the *rnd* mutation can restore development in OC. The presence of a larger Pxr-specific form (Pxr-XL) synchronized with the absence of Pxr-L in the *rnd* mutant suggests that Pxr processing happens sequentially and that there is at least one additional step (Pxr-XL to Pxr-L) in the processing pathway that occurs prior to the processing of Pxr-L to Pxr-S. Moreover, the observed in vitro processing of Pxr provides strong evidence that processing of Pxr-XL to Pxr-L depends on RNase D. Taken together, our findings refine the Pxr-mediated checkpoint model with an additional regulatory element governed by a ribonuclease known primarily for its housekeeping functions (Figure 5).

Although there are several reports of bacterial sRNA processing [34,35,36,37,38], the discovery of Pxr-XL is somewhat surprising, as it is only detectable in the *rnd* mutant, and it appears to be almost instantaneously processed into Pxr-L during both vegetative growth and development. This is similar to sequential processing events that produce mature eukaryotic miRNAs, where a primary transcript is quickly processed into a precursor form and the precursor is then processed into the mature miRNA [33]. The precursor miRNA has a 3′ overhang and a double-stranded stem allowing for recognition and transport to the cytoplasm [39]. It is unclear what functional role an analogous precursor sRNA might play in bacteria. The *rnd* mutants examined here do not appear to have any obvious defects that would hint at a role for Pxr-L.

Our results show that processing Pxr-XL to Pxr-L is mediated by RNase D, while the role of RNase D in processing Pxr-L to Pxr-S is inconclusive. In vivo complementation of *rnd* restores the presence of Pxr-L and Pxr-S, while the addition of RNase D in vitro only generates Pxr-L (Figure 4B). It is possible that processing Pxr-L to Pxr-S requires another ribonuclease that is absent in the in vitro assay. In most cases where bacterial sRNA processing occurs, an endoribonuclease, usually RNase E or RNase III, cleaves the mRNA [34,35,38,40,41]. However, our previous transposon- and spontaneous-mutation screens did not identify either of these ribonucleases [24,26]. Our in vivo results suggest that a basal level of *rnd* expression under the vanillate promotor is sufficient to abolish the developmental proficiency of OC::TnE1 (Figure 4A). We also found that *rnd* expression levels are maintained at lower levels after 9 h of starvation in wild-type cells (Figure S2). If RNase D is responsible for processing Pxr-L to Pxr-S, then it is likely that the continued albeit lower expression of *rnd* in the wild-type genetic background upon starvation would be sufficient to produce Pxr-L and Pxr-S. However, no Pxr-S is detected after as little as 0.5 h of starvation [22] in wild-type cells. These results are consistent with there being a second ribonuclease that processes Pxr-L to Pxr-S.

Alternatively, RNase D may process Pxr-L to Pxr-S, but Pxr-S may be unstable on its own and require a stabilizing partner that is missing in the reconstituted in vitro assay. Davis et al. [35] also hypothesized that the processed form of an sRNA might need a partner to promote cleavage or sRNA stabilization after they found that processed MicX sRNA primarily accumulated in wild-type *V. cholera*, but in an *hfq* mutant background the processed form was not detectable.

A study focused on *M. xanthus* developmental signaling independently found that mutations in *rnd* were able to bypass a developmental defect caused by a mutation in *bsgA*, a gene necessary for the production of B-signal, one of the major signals necessary for *M. xanthus* development (Cusick, Hager, and Gill, 2015). The *bsgA* gene encodes a *lon* protease homolog (Lon D), and the authors suggested that the Lon D protease could remove a transcriptional repressor and therefore be required for turning on the downstream developmental genes. Given that RNase D is involved in Pxr sRNA processing, we speculate that RNase D and/or the putative RNase X (a hypothesized ribonuclease responsible for Pxr-L to Pxr-S processing, see Figure 5) might be substrates of Lon D. The reduction in RNase D and RNase X levels by Lon D activity early in the developmental program might contribute to eliminating Pxr-S sRNA, hence allowing development to proceed (Figure 5). In this scenario, by preventing Pxr-S accumulation, an *rnd* mutant would not require a functional *bsgA* gene for development. Interestingly, another two mutations bypassing the *bsgA* requirement for development are localized in the *spdS and spdR* genes [42,43,44]*,* also known as *pxrK and pxrR. pxrR* encodes a σ^54^ response regulator, while *pxrK* encodes the cognate histidine kinase, together comprising a two-component signaling system required for Pxr synthesis [24]. Our findings suggest a linkage between the B-signal protease Lon D and the sRNA Pxr. We hypothesize that the Pxr developmental checkpoint lies downstream of B-signaling in the developmental pathway and that the role of Lon D is to remove negative regulation by Pxr.

Enzymes with a shared ancestral function across many species can evolve new, specialized functions in only some species [45,46,47]. Our finding that one function of RNase D in the myxobacteria is the regulation of multicellular fruiting-body development by processing the negative regulator Pxr raises intriguing questions regarding how *rnd* evolved to play this role. A major goal of microbial evolutionary developmental biology—or ”microbial evo-devo” [48]—is to understand how the genetic programs underlying developmental processes evolved. Further study of the epistatic and pleiotropic relationships of *rnd* with other genes during the transition from growth to development will promote understanding of the evolutionary process by which *rnd* came to regulate myxobacterial development, as well as perform other functions shared beyond the myxobacteria.

## Figures and Tables

**Table 1:** Strains, primers, and plasmids.

Strain/Primer/Plasmid	Description	Reference
OC, also known as GVB207.3	Developmentally defective descendent of GJV1	[30]
OC::TnE1	*MXAN*_*5981* transposon-insertion mutant of OC, developmentally proficient	[26]
OC::TnE1-r	Reconstructed OC::TnE1 transposon mutant	This study
OC∆*pxr*	*pxr* deletion mutant of OC	[22]
GJV1∆*pxr*	*pxr* deletion mutant of GJV1	[22]
OC::TnE1pMR3629*rnd*	OC::TnE1 containing *MXAN*_*5981* under a vanillate-inducible promotor.	This study
OC::*MXAN*_*5982*	*MXAN*_*5982* plasmid-interruption mutant	This study
GJV1	Laboratory wild-type, developmentally proficient, evolutionary ancestor of OC	[30]
GJV1::TnE1	Reconstructed GJV1 transposon-insertion mutant	This study
M13	5′GCCAGGGTTTTCCCAGTCACGA3′	
M13r	5′GAGCGGATAACAATTTCACACAGG3′	
GV545	5′CCCGGTCCGCTAGCCTGCCTCCC3′	This study
GV619	5′;TACACGATGACCCAGTTCCA3′	This study
GV620	5′GACTCGTTGAGCCAGAGGTC3′	This study
GV700	5′GGTACGCGTAACGTTCGAATTC3′	This study
GV713	5′TTTTCATATGCCGACCTACCCGCAAGGTGCCG3	This study
GV714	5′TGCCGGCCCCACCTTCACTTCAGGCG3	This study
GV716	5′TCTCGAGACATCATCATCATCATCATATGCCGACCTACCCGC3′	This study
pCR-*rnd*	pCR-Blunt containing the *rnd* insertion sequence	This study
pMR3629-*rnd*	pMR3629 containing the *rnd* insertion sequence	This study
pCR-5982	pCR-Blunt containing the *MXAN_5982* insertion sequence	This study
pBAD-his-*rnd*	Overexpression plasmid for histidine-tagged *rnd*	This study
pMR3629	Plasmid containing a vanillate-inducible promotor and oxytetracycline resistance	[31]
pCR-Blunt	Invitrogen cloning vector	Invitrogen
pBAD-*Myc*-HisA	Invitrogen protein expression vector	Invitrogen

## Data Availability

The data supporting the findings of this study are available within the article and its Supplementary Materials.

## Supplementary Materials

The following supporting information can be downloaded at: https://www.mdpi.com/article/10.3390/genes14051061/s1, Figure S1: Northern blot showing Pxr-S; Figure S2: The rnd transcript quantity decreases upon starvation.
