# Supplementary material for: Ribonuclease D Processes a Small RNA Regulator of Multicellular Development in Myxobacteria
Source: Genes (Basel). 2023 May 9;14(5):1061. doi: 10.3390/genes14051061 (PMC10217877; doi:10.3390/genes14051061)
Supplement: Supplementary file 1 [file genes-14-01061-s001.zip › Figure S1 and Figure S2.pdf]

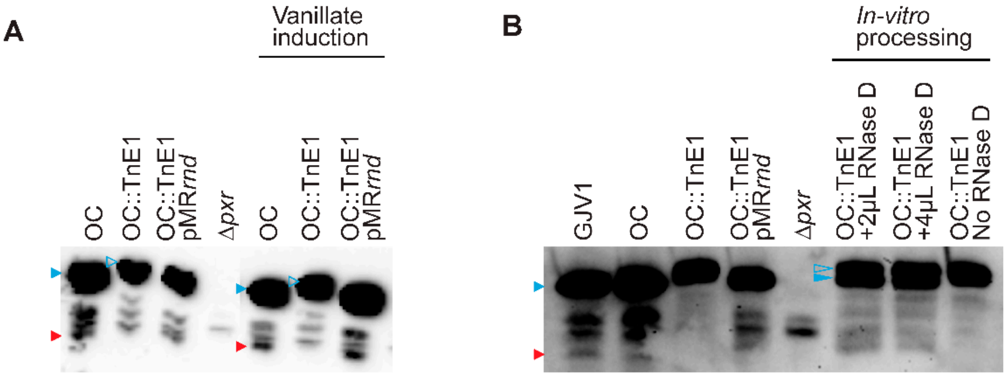

Figure S1. Northern blot showing Pxr-S. Longer exposure times of the *in vivo* and *in vitro* Northern blots shown in Figure 4 allow better visualization of Pxr-S but reduce visualization of the distinction between Pxr-XL and Pxr-L. The Northern blots shown for (A) and (B) are the same as for Fig. 4 except with longer exposure. Arrow designations are the same as in Fig. 4.

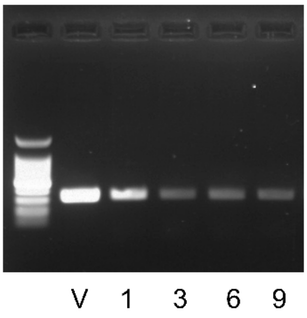

Figure S2. *rnd*-transcript quantity decreases upon starvation. Comparison of *rnd*-transcript levels after rt-PCR amplification with *rnd*-specific primers for samples isolated from strain GJV1 at the vegetative mid-log state (V), and 1, 3, 6 and 9 hours post-starvation.
